# Reduced Susceptibility to Colitis-Associated Colon Carcinogenesis in Mice Lacking Plasma Membrane-Associated Sialidase

**DOI:** 10.1371/journal.pone.0041132

**Published:** 2012-07-17

**Authors:** Kazunori Yamaguchi, Kazuhiro Shiozaki, Setsuko Moriya, Koichi Koseki, Tadashi Wada, Hiroo Tateno, Ikuro Sato, Masahide Asano, Yoichiro Iwakura, Taeko Miyagi

**Affiliations:** 1 Division of Molecular and Cellular Oncology, Miyagi Cancer Center Research Institute, Natori, Japan; 2 Laboratory of Marine Biochemistry, Faculty of Fisheries, Kagoshima University, Kagoshima, Japan; 3 Division of Cancer Glycosylation Research, Institute of Molecular Biomembrane and Glycobiology, Tohoku Pharmaceutical University, Sendai, Japan; 4 Division of Pathology, Miyagi Cancer Center Research Institute, Natori, Japan; 5 Surgical Pathology Japan, Inc., Sendai, Japan; 6 Division of Transgenic Animal Science, Advanced Science Research Center, Kanazawa University, Kanazawa, Japan; 7 Center for Experimental Medicine and Systems Biology, Institute of Medical Science, University of Tokyo, Tokyo, Japan; Charité, Campus Benjamin Franklin. Germany

## Abstract

Sialic acids are acidic monosaccharides that bind to the sugar chains of glycoconjugates and change their conformation, intermolecular interactions, and/or half-life. Thus, sialidases are believed to modulate the function of sialoglycoconjugates by desialylation. We previously reported that the membrane-associated mammalian sialidase NEU3, which preferentially acts on gangliosides, is involved in cell differentiation, motility, and tumorigenesis. The NEU3 gene expression is aberrantly elevated in several human cancers, including colon, renal, prostate, and ovarian cancers. The small interfering RNA-mediated knock-down of NEU3 in cancer cell lines, but not in normal cell-derived primary cultures, downregulates EGFR signaling and induces apoptosis. Here, to investigate the physiological role of NEU3 in tumorigenesis, we established *Neu3*-deficient mice and then subjected them to carcinogen-induced tumorigenesis, using a sporadic and a colitis-associated colon cancer models. The *Neu3*-deficient mice showed no conspicuous accumulation of gangliosides in the brain or colon mucosa, or overt abnormalities in their growth, development, behavior, or fertility. In dimethylhydrazine-induced colon carcinogenesis, there were no differences in the incidence or growth of tumors between the *Neu3*-deficient and wild-type mice. On the other hand, the *Neu3*-deficient mice were less susceptible than wild-type mice to the colitis-associated colon carcinogenesis induced by azoxymethane and dextran sodium sulfate. These results suggest that NEU3 plays an important role in inflammation-dependent tumor development.

## Introduction

Sialic acids are widely distributed across species, from viruses and microorganisms to higher animals, including all mammals [1]. They usually exist as glycoconjugate-bound forms, and the bound sialic acids can modify the conformation, intermolecular interactions, and half lives of the glycoconjugates. Many reports have shown that the sialylation level of glycoconjugates changes during physiological and pathological processes such as cell growth, differentiation, immune responses, and tumorigenesis [2]. For example, upon malignant transformation, the amounts and types of sialic acids on cell-surface glycoproteins or glycolipids are altered, although the physiological meanings of these changes have not been fully elucidated [3]–[5].

As expected from the effects on glycoconjugates of binding sialic acids, the removal of sialic acid moieties also affects biological processes. Sialidases (EC 3.2.1.18; acetylneuraminyl hydrolase) are alpha ketosidases that catalyze the removal of sialic acids from glycoconjugates. In mammalian cells, there are four sialidases that differ in their subcellular localizations, substrate preferences, optimum pH, and sensitivity to inhibitors [2], [6]. These distinct characteristics may reflect the sialidases' different physiological roles. Of the four mammalian sialidases, NEU3 localizes to the membrane fraction of cells and shows a strong preference for gangliosides as substrates [7], [8].

We and others have shown that NEU3 can modulate biological processes, including neuronal differentiation [9]–[11], T-cell activation [12], monocyte differentiation [13], cell adhesion and motility [14], [15], and the onset of a diabetic phenotype [16]. In addition, we demonstrated that human NEU3 is upregulated in terms of both enzymatic activity and mRNA level in many human cancers, including colon [17], renal [18], prostate [19], and ovarian [20] cancers. NEU3 appears to be indispensable for the survival of cancer cells, since small interfering RNA-mediated knock down of *NEU3* in cancer cell lines leads to decreased epidermal growth factor receptor (EGFR) phosphorylation and the suppression of Ras and extracellular signal-regulated kinase (ERK) activation, which in turn results in apoptosis of the cells [21]. Interestingly, *NEU3* knock down did not induce apoptosis in similarly treated primary cultures of fibroblasts or keratinocytes. Human *NEU3-*expressing transgenic mice, whose colon mucosa had 33-times more sialidase activity than that of wild-type mice, showed enhanced formations of aberrant crypt foci (ACF) in response to the colonogenic carcinogen, azoxymethane (AOM) [22]. These results suggested that NEU3 has a role in tumor development. To examine this possibility under physiological conditions, in the present study we generated *Neu3*-deficient mice and analyzed their susceptibility to tumorigenesis in carcinogen-induced models of sporadic and colitis-associated colon cancer. We found that *Neu3*-deficiency lowered the incidence of colitis-associated colon carcinogenesis.

## Materials and Methods

### Chemicals

AOM was purchased from Wako Pure Chemicals (Osaka, Japan). Dextran sulfate sodium salt (DSS) (molecular weight of 40,000) was from ICN Biochemicals (Aurora, OH). Other reagents were purchased from Sigma unless otherwise specified.

### Targeting vector construction

**Figure 1:**
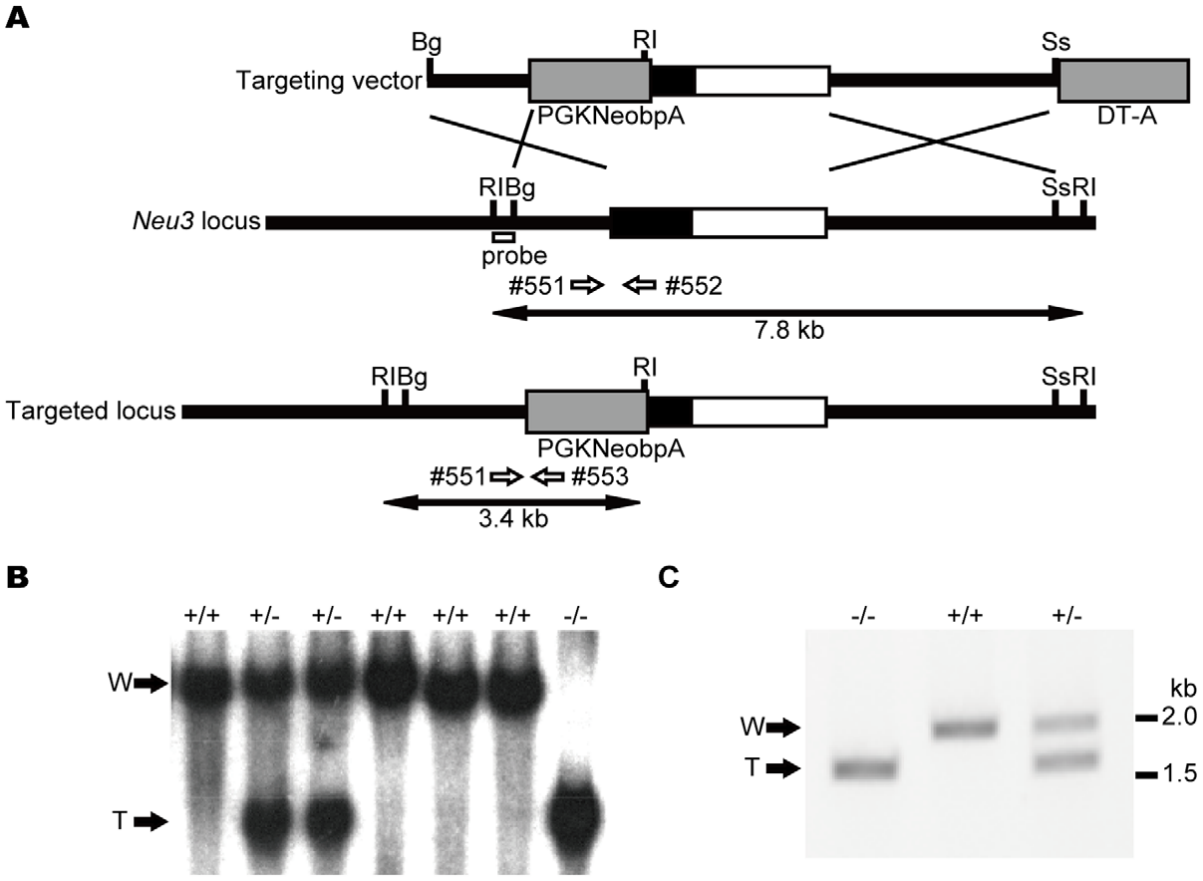
Targeting of the mouse *Neu3* gene. A. Targeting strategy. Exons and introns are indicated by boxes and lines, respectively. Open boxes and filled boxes indicate non-coding and coding exons, respectively. Expression cassettes for the PGK-driven Neo gene (PGKNeobpA) and for diphtheria toxin A (DT-A) are indicated by thick gray boxes. The site of the probe for Southern blotting is indicated by a small open box. The sites of PCR primers are indicated by open arrows. Bg: BglII, RI: EcoRI, SS: Sse8387I. B. Southern blotting analysis of mice generated by crossing *Neu3* heterozygous mice. Genomic DNA was prepared from a tail chip, digested by EcoRI, and analyzed by Southern blotting using the probe indicated in A. DNA fragments from the wild (W) and targeted (T) allele are indicated by arrows. C. PCR analysis of the homologous recombination in *Neu3*-deficient mice. Amplified DNA fragments from the wild (W) and targeted (T) allele are indicated by arrows.

A bacterial artificial chromosome (BAC) library clone containing the mouse *Neu3* gene was purchased from Genome Systems (St Louis, MO) and used to construct the targeting vector. A 7-kb fragment harboring exon 3 of the gene was excised from the BAC clone DNA and subcloned into pBluescriptII SK(+) (Stratagene). A PGKneobpA cassette [23] was inserted between the SpeI and NcoI sites of the *Neu3* exon 3 to disrupt the coding region and enable positive selection. A DT-A cassette was inserted into the 3′ end of the targeting vector for negative selection [24] (Fig. 1).

### Generation of *Neu3*-deficient (*Neu3* ^−/−^) mice

The targeting vector was linearized by digestion with KpnI and electroporated into R1 embryonic stem (ES) cells [25] as described previously [26]. Homologous recombinants of the ES cells were screened by nested polymerase chain reaction (nested PCR) using Z-Taq polymerase (Takara Bio, Otsu, Japan). The first PCR was carried out at 95°C for 5 min for the initial denaturation followed by 32 cycles of 98°C 1 sec, 60°C 10 sec, and 72°C 20 sec with primers ATGGCTAGGCGTGTGCTCTACCCCATTC and GCCTGCTTGCCGAATATCATGGTGGAAAAT. The resulting PCR products were used as a template for the second PCR, using the primers GCTCTACCCCATTCTACATCTCCAGAC and TCGTGCTTTACGGTATCGCCGCTCCCGATT, under the same conditions as for the first PCR.

Chimeric mice were generated as described before [26], mated with C57BL/6J for 14 generations, and then intercrossed for more than 20 generations. Genotypes were determined by Southern blotting with standard techniques [27] or by PCR with KOD FX polymerase (Toyobo, Japan) and primers #551 (GCTCTACCCCATTCTACATCTCCAGAC), #552 (TCGTGCTTTACGGTATCGCCGCTCCCGATT), and #553 (GTGAGTTCAAGAGCCATGTTGCTGATGGTG). PCR was carried out at 94°C for 4 min for the initial denaturation followed by 30 cycles of 98°C 10 sec and 68°C 150 sec. Primers #551 and #552 generated a DNA fragment of 2110 bp derived from the wild-type allele, whereas primers #551 and #553 generated a DNA fragment of 1689 bp derived from the targeted allele. Mice were fed a pelleted basal diet (CRF-1, Oriental Yeast, Tokyo, Japan) and water *ad libitum,* and were maintained under a 12-h light and 12-h dark cycle. The mice were all subjected to experimentation at 6 to 7 weeks of age. All animal experiments were approved by the Animal Care Committee of Miyagi Cancer Center.

### Reverse transcription (RT)-PCR

The levels of transcripts for mouse sialidases were evaluated by quantitative RT-PCR as described previously with minor modifications [28]. Total RNA was prepared from mouse tissues using an RNeasy mini kit (Qiagen) and reverse transcribed with PrimeScript (Takara), according to the manufacturer's recommendations. Real-time PCR was performed with a QuantiTect SYBR Green PCR kit (Qiagen) and Light Cycler PCR system (Roche). Samples were subjected to denaturation at 94°C for 15 min followed by 45 cycles of 94°C 15 sec, 60°C 30 sec, and 72°C 30 sec. The primers used were TTCATCGCCATGAGGAGGTCCA and AAAGGGAATGCCGCTCACTCCA for *Neu1*, AGGAAGCTACAACGAAGCCACA and TTCTGAGCAGGGTGCAGTTTCC for *Neu2,* CTCAGTCAGAGATGAGGATGCT and GTGAGACATAGTAGGCATAGGC for *Neu3*, and AGGAGAACGGTGCTCTTCCAGA and GTTCTTGCCAGTGGCGATTTGC for *Neu4*. For the normalization of sample variation, the transcript level of glyceraldehyde-3-phosphate dehydrogenase (*Gapdh*) was measured in parallel using the primers ACCACAGTCCATGCCATCAC and TCCACCACCCTGTTGCTGTA. A standard curve for each target gene was generated by serial dilution of the pBluescript vector containing the open reading frame (ORF) of the respective gene [28].

### Sialidase activity assays

Samples for activity assays were extracted from the brain or colon mucosa by sonicating the tissue in buffer (50 mM Hepes [pH 7.5], 150 mM NaCl, 1% Nonidet P40, 2 mM Ethylenediamine-N, N, N', N'-tetraacetic acid [EDTA], 7.5 μg/mL aprotinin, 10 μg/mL leupeptin, 10 mM NaF, 2 mM orthovanadate, 0.25% sodium deoxycholate, and 2 mM phenylmethylsulfonyl fluoride [PMSF]). The sialidase activity, using GM3 or 4-methylumbelliferyl N-acetylneuraminic acid (4MU-NeuAc) as the substrate, was measured as described previously [7]. Protein concentrations were determined by a dye-binding assay (Bio-Rad Laboratories) performed according to the manufacturer's recommendations.

### Glycolipid preparation and high performance thin-layer chromatography (HPTLC)

Preparation and HPTLC analysis of glycolipids were performed as described previously [18]. In brief, total lipids were obtained by the sequential extraction of brain or colon mucosa with chloroform/methanol (1∶1 and then 1∶2, v/v). The extracts were combined and saponified with 0.1 N NaOH in methanol. The saponified lipids were desalted with a Sep-Pak C_18_ cartridge (Waters) and then applied to a DEAE-A25 column (GE-Healthcare) equilibrated with chloroform/methanol/water (30:60:8, v/v/v). The column was washed with the same solvent. The flow-through and wash fractions were collected and pooled as the neutral glycolipids fraction. The acidic glycolipids were eluted from the washed column with chloroform/methanol/0.13 M ammonium acetate (30:60:8, v/v/v) and desalted with a Sep-Pak C_18_ cartridge. The neutral and acidic glycolipids were then applied to an HPTLC plate (Merck) and fractionated with chloroform/methanol/0.2% CaCl_2_ (50:40:5, v/v/v). The fractionated glycolipids were visualized with orcinol-H_2_SO_4_ or resorcinol-HCl.

### Aberrant crypt foci (ACF) induction and analysis

For ACF induction, AOM or dimethylhydrazine (DMH) was used as a carcinogen. AOM was diluted with sterile saline solution (0.9% NaCl solution) and injected intraperitoneally into mice once a week for 2 weeks at a dose of 10 mg/kg body weight. Five weeks after the first injection, the colons were dissected, flushed with calcium- and magnesium-free phosphate-buffered saline (PBS(-)), cut open longitudinally, placed flat on filter paper, and fixed in 10% buffered formalin for 48 h. The fixed colons were then stained with 0.2% methylene blue, and the ACF were scored as previously described [22]. The ACF were distinguished from the surrounding normal crypts by their larger size, thicker epithelium, and deformed luminal opening [29]. DMH was diluted with sterile saline, adjusted to pH 6.5 with NaOH, and then injected intraperitoneally into mice once a week for 10 weeks at a dose of 20 mg/kg body weight. Fourteen weeks after the first injection, the colons were dissected and processed for ACF observation as described above.

### Tumor induction and analysis

For the sporadic colon carcinogenesis model [30], mice were intraperitoneally injected with DMH (30 mg/kg body weight) once a week for 20 weeks, and 4 weeks after the last injection, the mice were killed by CO_2_ asphyxiation and examined for colon tumor development. For the colitis-associated carcinogenesis model [31], mice were given a single intraperitoneal injection of AOM at a dose of 10 mg/kg body weight, and one week later 1.5% DSS was given in the drinking water for 7 days. The mice were sacrificed 20 weeks after the AOM injection. The colons were removed and fixed as described above. The tumors were counted and measured by caliper under microscopic observation. To calculate the tumor volume, the modified ellipsoid formula, 1/2(Length × Width^2^), was used [32], [33].

### Statistics

The results are expressed as means ± SD. The non-parametric Mann-Whitney *U* test was used for all pair-wise comparisons.

## Results

### Generation of *Neu3*-deficient (*Neu3* ^−/−^) mice

To investigate the physiological role of NEU3 sialidase in tumorigenesis, we established mice with a disrupted *Neu*3 gene. To inactivate the *Neu3* gene, a targeting vector was designed to delete part of exon3 (Fig. 1A). The vector was electroporated into ES cells, and the correctly targeted ES cells were confirmed by PCR and used to generate chimeric mice that transmitted the disrupted alleles to their offspring, as described in *Materials and Methods*. The intercrossing of heterozygous (*Neu3* ^+/−^) mice produced viable homozygous *Neu3*-deficient (*Neu3* ^−/−^) mice (Fig. 1B, C) at the expected ratio, indicating there was no embryonic lethality in the *Neu3*-deficient mouse.

**Figure 2:**
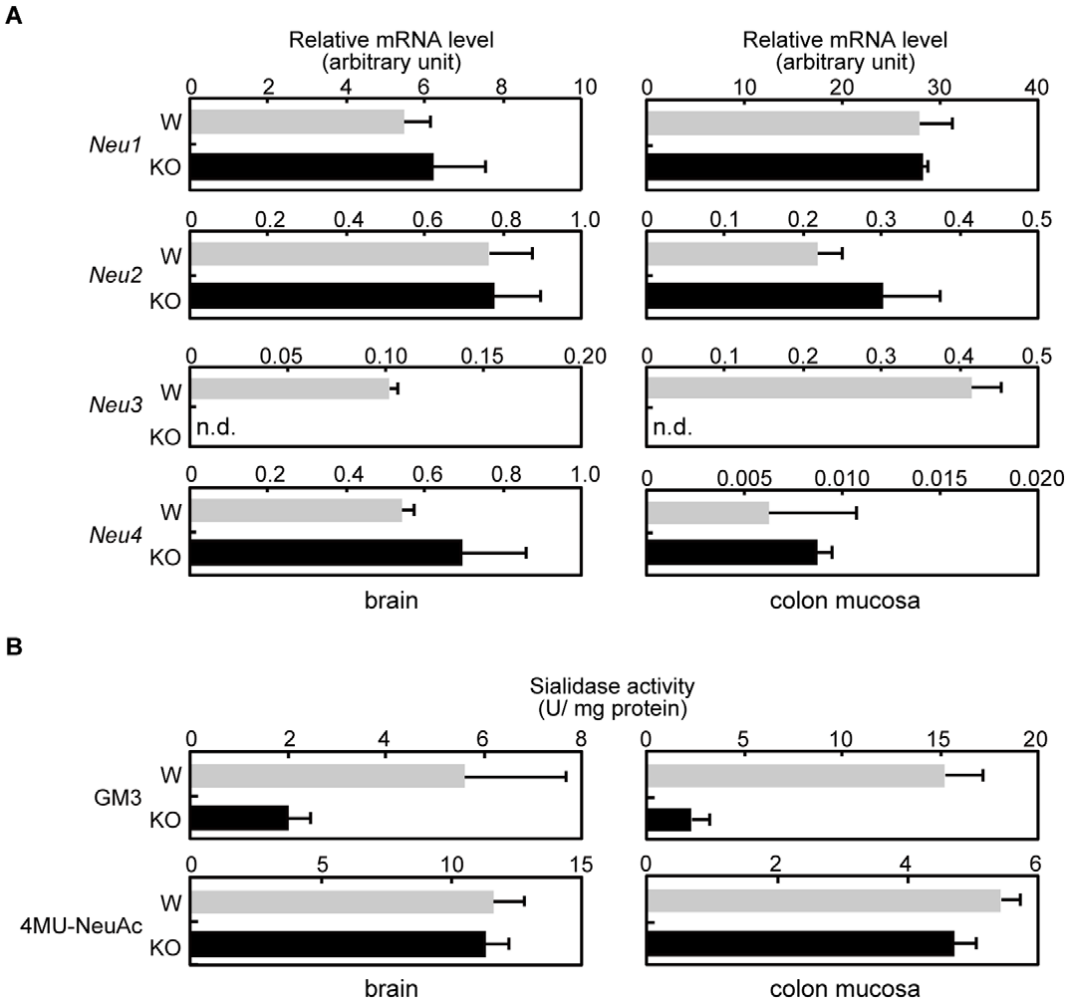
Gene expression and activity of sialidases in *Neu3*-deficient mice. A. Relative mRNA levels of sialidases in the brain (left panels) or colon mucosa (right panels) of wild-type (W, gray bars) or *Neu3*-deficient (KO, filled bars) mice. RNA preparation, reverse transcription, and quantitative PCR were performed as described in *Materials and Methods*. For quantification, a standard curve for each target gene was generated by serial dilution of the plasmid DNA containing the ORF of the respective gene. Results are averages obtained from three mice of each genotype. The RNA level of each sialidase was normalized to that of *G3pdh* and expressed in arbitrary units. n.d.: not detected. B. Relative sialidase activity of the brain (left panels) or colon mucosa (right panels) of wild-type (W, gray bars) or *Neu3*-deficient (KO, filled bars) mice. Crude enzyme was prepared and assayed as described in *Materials and Methods*. Enzyme assays using GM3 (upper panels) or 4MU-NeuAc (lower panels) as the substrate are shown. Results are averages obtained from three mice of each genotype. Activity is expressed in units/mg protein. One unit was defined as the amount of enzyme that cleaved 1 nmol of sialic acid per hour.

To confirm the deficiency of *Neu3* gene expression, we performed RT-PCR to detect its mRNA. As shown in Fig. 2A, the mRNA of *Neu3* was under the detectable level in the brain and colon mucosa. The mRNA levels of other mouse sialidases *(Neu1*, *Neu2*, and *Neu4*) showed no difference between the wild-type and *Neu3*-deficient mice, indicating that only the *Neu3* gene was inactivated. Interestingly, the colon mucosa of wild-type mice showed substantial *Neu3* expression, but the human colon mucosa shows no or only faint expression of *NEU3* [17], suggesting that the expression of *Neu3* genes are differently regulated in mice versus humans.

To assess the sialidase activities of the *Neu3*-deficient mice, we measured the activities in crude extracts prepared from the brain or colon mucosa using ganglioside GM3 or 4-methylumbelliferyl-neuraminic acid (4MU-NeuAc), a good or a poor substrate for NEU3, respectively (Fig. 2B) [8], [9]. The sialidase activity of the *Neu3*-deficient mice for GM3 was decreased in both tissues, whereas the activity for 4MU-NeuAc was scarcely affected, also suggesting that only the *Neu3* gene was inactivated.

The brain of the *Neu3*-deficient mice showed residual GM3-hydrolyzing activity, which might be explained by the dominant expression of NEU4 sialidase in mouse brain [34], [35]. This residual activity seemed to be sufficient for maintaining ganglioside homeostasis, because the analysis of brain gangliosides in the *Neu3*-deficient mice revealed no significant differences when compared with those in wild-type mice (Fig. S1A). We also could not detect any clear difference in ganglioside composition in the colon mucosa in this study (Fig. S1B). These results suggest that the absence of NEU3 has little effect on ganglioside catabolism in the brain or colon mucosa of *Neu3*-deficient mice, presumably because of compensation by NEU4 or by the modulation of certain ganglioside-synthesizing enzyme(s), as shown in previous siRNA-mediated knock-down studies [21], [36]. On the other hand, we cannot completely exclude the possibility of changes in one or more minor gangliosides that could not be clearly detected by our TLC analysis.

*Neu3*-deficient mice lived for one year or longer, similar to wild-type mice, and showed no obvious abnormalities in their appearance or fertility. Histological analyses revealed no obvious differences between the *Neu3*-deficient and the wild-type mice in the brain, liver, heart, testis, kidney, lung, or muscle (data not shown). We also established another *Neu3*-deficient mouse line on a Balb/c background and found no overt abnormalities in this line either (data not shown).

### Susceptibility of *Neu3*-deficient mice to colitis-associated colon tumor

To examine NEU3's involvement in tumorigenesis, we first analyzed the ACF formation induced by alkylating reagent AOM or DMH. ACF are putative premalignant lesions of colon cancers in rodents and humans [37] and can be induced by carcinogen treatment in rodents [29]. Several studies have shown that the incidence and multiplicity (number of crypts per focus) of ACF is generally correlated with the incidence and growth of colon tumors, respectively [38]. We previously observed a 2-fold greater incidence of AOM-induced ACF formation in transgenic mice expressing high levels human *NEU3* than in wild-type mice [22]. Here we administered AOM to *Neu3*-deficient and wild-type mice by intraperitoneal injection, and colon specimens were obtained and subjected to ACF counting as described in *Materials and Methods*. All the AOM-treated mice developed ACF, and the *Neu3* deficiency did not affect the ACF numbers (wild-type vs. KO: 41.8±3.8 vs. 40.0±11.4, n = 4/group, Fig. S2A) or multiplicity (wild-type vs. KO: 2.0±1.0 vs. 2.2±1.0, data not shown). Similar results were obtained using DMH instead of AOM as the carcinogen: the ACF numbers (wild-type vs. KO: 37.7±9.8 vs. 42.0±17.1, n = 6/group, Fig. S2B) and multiplicity (wild-type vs. KO: 2.2±0.9 vs. 2.1±0.8, data not shown) showed no differences between the *Neu3*-deficient and the wild-type mice. These results suggest that AOM- or DMH-induced ACF formation could proceed without *Neu3* in this experimental system.

**Figure 3:**
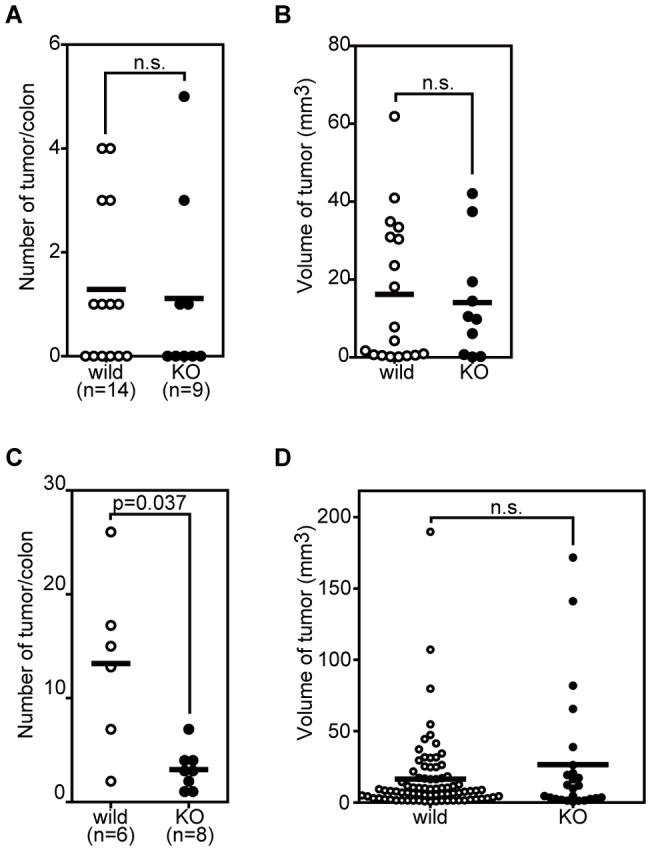
Carcinogen-induced tumorigenesis in the colon of *Neu3*-deficinet mice. *Neu3*-deficient mice or wild-type mice were subjected to multiple injections of DMH (A, B) or a single AOM injection followed by DSS administration in the drinking water (C, D) as described in *Materials and Methods*. The number (A, C) and volume (B, D) of tumors were determined by microscopic observation. Horizontal bars indicate the mean tumor number and volume. Statistical significance was assessed by non-parametric Mann-Whitney *U* test. n.s.: not significant.

**Figure 4:**
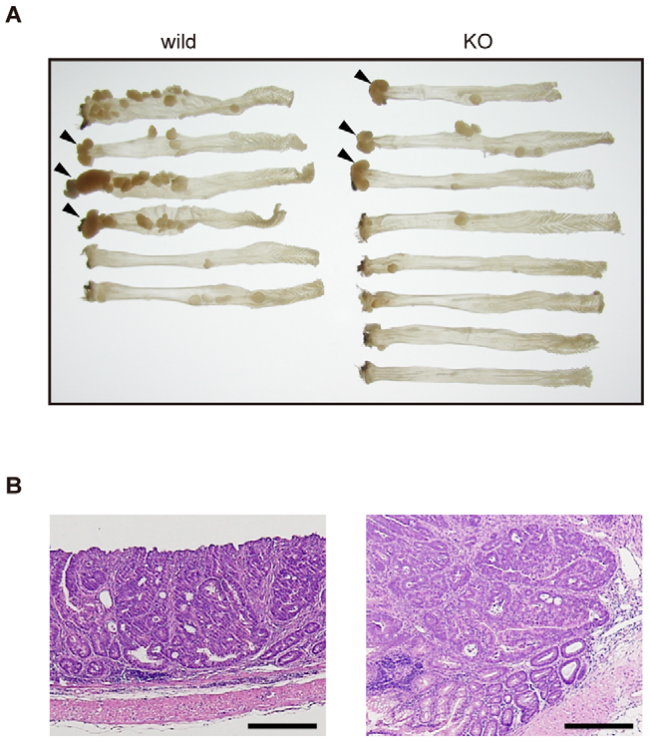
Colon tumors induced by AOM/DSS treatment. *Neu3*-deficient or wild-type mice were subjected to a single AOM injection followed by DSS administration in the drinking water as described in *Materials and Methods.* Colons were dissected 20 weeks after AOM injection and fixed with formalin. A. Macroscopic photograph of dissected colons. Tumors developed at the distal ends of colons are indicated by arrowheads for easy recognition. B. Micrographs of the colon adenocarcinoma. Representative examples of adenocarcinomas developed in the *Neu3*-deficinet (left) or in the wild-type (right) mouse. Sections from paraffin-embedded tumors were stained with hematoxylin and eosin by standard techniques. Bars, 0.1 mm.

We next subjected the *Neu3*-deficient mice to two established colon tumorigenesis protocols [30]: one used multiple intraperitoneal injections of AOM or DMH to examine sporadic tumor development in mice, and the other used a single AOM or DMH injection followed by administration of the inflammatory reagent DSS, to examine colitis-associated colon carcinogenesis [31]. For the sporadic model, we used DMH instead of AOM, because multiple injections of AOM were highly lethal for the mice in our experiments. Inspection of the dead animals suggested that they had severe hepatitis (data not shown) [39]. The *Neu3*-deficient mice and the wild-type mice received a weekly injection of DMH for 20 weeks, and 4 weeks after the last injection, the mice were killed and examined for colon tumor incidence and size. As shown in Fig. 3A, the DMH-treated *Neu3*-deficient mice developed colon tumors at an incidence (4/9, 44%) comparable to that of the wild-type mice (8/14, 57%). In addition, no significant difference was found in the tumor multiplicity (wild-type vs. KO: 2.3±1.4 vs. 2.5±1.9, data not shown) or the tumor volumes (Fig. 3B) between the *Neu3*-deficient and the wild-type mice. Histological analysis of the tumors revealed that they were all adenocarcinomas, and no differences between the two genotypes were found (data not shown). For the colitis-associated colon carcinogenesis model, the mice were given a single intraperitoneal injection of AOM followed by 1.5% DSS in the drinking water for 1 week. The water intake was the same for the two genotypes (data not shown). Twenty weeks after the AOM injection, the tumor incidence and volume were examined as described in *Materials and Methods*. All the mice treated with AOM/DSS developed colon adenocarcinoma (Fig. 4), but the number of adenocarcinomas per mouse was significantly lower in the *Neu3*-deficient mice than in the wild-type mice (Fig. 3C, p = 0.037), suggesting NEU3 is involved in colitis-associated colon carcinogenesis. Two other preliminary experiments using small numbers of mice also suggested that *Neu3*-deficient mice had a decreased susceptibility to colitis-associated carcinogenesis (Fig. S3). There was no statistically significant difference in tumor sizes, however, between the *Neu3*-deficient and the wild-type mice (Fig. 3D, p = 0.734).

To elucidate molecular mechanisms of decreased susceptibility to colitis-associated colon carcinogenesis in *Neu3*-deficient mice, we analyzed EGFR signaling in colon mucosa of DSS-treated mice as described previously [22], because EGFR signaling is involved both in colitis-associated colon carcinogenesis [40] and NEU3-induced formation of ACF [22]. We analyzed activation levels of signaling molecules EGFR and AKT but could not detect any significant differences between the *Neu3*-deficient and the wild-type mice (data not shown). Neither could we find any differences in expression of inflammation-related genes, *Il-6*, *Il-23*, and *Cox2* (data not shown) between the *Neu3*-deficient and the wild-type mice. Thus underlying mechanism connecting *Neu3*-deficiency and reduced susceptibility to colitis-associated colon carcinogenesis remains to be elucidated.

## Discussion

Our present findings show that the loss of the *Neu3* gene in mice lowered the incidence of colitis-associated colon carcinogenesis, whereas the loss had no apparent effect on tumor incidence or growth in a sporadic colon carcinogenesis model. These results suggest that NEU3 has a physiological role(s) in inflammation-related carcinogenesis, which has not hitherto been addressed.

Accumulating evidence suggests that inflammation has important roles in carcinogenesis. A spectrum of cytokines, prostaglandins, and reactive oxygen species are induced upon inflammatory stimuli and act on tumor or stromal cells, resulting in enhanced growth or survival of the tumor cells [41]. In addition, inflammation-related signaling pathways such as NF-κB have been shown to be important for tumor cell growth and survival [42]. Clinical research has also revealed an upregulation of inflammatory signaling not only in leukemia but also in many types of human solid tumors [43]. Although the molecular mechanism(s) linking NEU3 function and inflammation remains totally unknown, our present study suggests that they are linked. We and others have shown that NEU1 [44], [45] and NEU4 [46] are involved in inflammatory responses, and in this context, studies on sialidases might contribute new insights into the regulation of inflammation under pathological conditions.

We previously revealed a requirement of cancer cells for NEU3 *in vitro*: the siRNA-mediated knock down of NEU3 induces the apoptosis of human cancer cells including colon cancer cell lines accompanied by suppression of the EGFR signaling pathway [21]. In the present study, however, we found that colon tumors still formed in mice in spite of the *Neu3* deficiency. Although this discrepancy remains to be elucidated, there are at least four possible explanations. One possibility is that the tumor microenvironment [47] attenuates the apoptosis induced by *Neu3*-deficiency *in vivo*. A second possibility is that mechanisms controlling cell growth and survival might have adapted to the *Neu3*-deficient conditions to allow tumor formation. Regarding this possibility, several reports have indicated that the constitutive or transient activation/inactivation of certain genes or signaling pathways can have different effects on cell growth or tumor formation [48], [49]. The third possibility is that susceptibilities to tumorigenesis are different between human and mouse cells [50], [51], presumably because of divergence in cell growth controls [51]–[54]. Mice might be too prone to tumorigenesis to reproduce the protective effect of NEU3 seen in human cancer cells. The fourth possibility is that the roles of NEU3 sialidase in colon tumorigenesis are different between human and mouse. We previously reported differences in enzymatic properties of mouse and human NEU3. Although the amino acid sequences of mouse and human NEU3 show 67.6% sequence identity and both NEU3s show high activities on gangliosides including GD3, the mouse NEU3 can hydrolyze ganglioside GM2 and sialyllactose (hydrolysis rates relative to GD3 are 14% and 30%, respectively) whereas the human NEU3 can scarcely hydrolyze these substrates (2% and 5%, respectively) under *in vitro* enzymatic assay [9]. Besides, the enzymatic activity of mouse NEU3 peaks at around pH 4.6 [9], whereas that of human NEU3 peaks at pH 4.5–4.8 and at pH 6.0–6.5 [55]. When expressed in cultured cells, mouse NEU3 localized at plasma membrane whereas human NEU3 localized at intracellular membraneous fractions as well as at plasma membrane ([15], Akita, Yamaguchi, and Miyagi, unpublished data). These differences in the enzymatic properties imply the different role(s) of these two enzymes [56]. Furthermore, as shown in Fig. 2A, mouse *Neu3* was expressed at a substantial level in the colon mucosa whereas human *NEU3* is not [17], further suggesting different roles of these two sialidases in the colon mucosa under physiological and presumably pathological conditions, including tumorigenesis.

In this study, we showed that the sialidase activity for gangliosides was decreased in brain extracts of *Neu3*-deficient mice, but the total levels of gangliosides in the brain showed little if any detectable variation from the wild-type case upon TLC analysis. This finding suggests that a sialidase(s) other than NEU3 is responsible for ganglioside homeostasis and that NEU3 activity does not make a major contribution to homeostasis. Our previous study *in vitro* implied that NEU3 could be the key enzyme for ganglioside homeostasis because this enzyme preferentially act on gangliosides whereas other three sialidases show more broad substrate specificities *in vitro*: they can act more effectively on glycoproteins, oligosaccharides, and synthetic substrate (4MU-NeuAc) and less effectively on gangliosides than NEU3 [2]. NEU1 was another sialidase considered to participate in ganglioside homeostasis since this enzyme exists abundantly in cells, which might circumvent its relatively lower activity toward gangliosides. Knock out mice studies, however, suggested that neither NEU3 nor NEU1 are major responsible enzymes for ganglioside degradation *in vivo*. The deficiency of *Neu3* or *Neu1* does not cause an aberrant accumulation of gangliosides in mice (this study and ref [57]). Instead, Seyrantepe et al showed that GD1a increases and GM1 decreases in *Neu4*-deficient mice, accompanied by the decreased sialidase activity in brain extracts [58]. Considering these observations and the dominant expression of *Neu4* in the mouse brain [34], [35], NEU4 may play a major role in ganglioside degradation in the mouse brain. The NEU3 activity might be restricted to degradation processes occurring within membranes under physiological conditions [59], [60]. Further studies using knock out mice for each sialidase would reveal *in vivo* substrates and bring progress in understanding metabolism of sialoglycoconjugates [61]. On the other hand, our previous results suggested a NEU3's function other than ganglioside metabolism: NEU3 might function as a signaling molecule by interacting with other signaling molecules, including EGFR, Grb2, or Rac1 [2]. The physiological roles of the NEU3, including its specific function in colitis-associated colon carcinogenesis, remain important topics for future study.

## Supporting Information

Figure S1 **Glycolipid patterns in** ***Neu3*** **-deficient mice.** A. Acidic glycolipids were extracted from the brain of the *Neu3*-deficient or wild-type mice and analyzed by TLC as described in *Materials and Methods*. The TLC plates were sprayed with resorcinol-HCl or orcinol-H_2_SO_4_ to visualize sialic acid-containing glycolipids or total glycolipids, respectively. Positions of standard gangliosides are indicated by bars. The bands indicated by asterisks were detected by orcinol staining but not by resorcinol staining, suggesting that they were contaminating neutral glycolipids. B. Glycolipids were extracted from the colon mucosa of the *Neu3*-deficinet and wild-type mice. Pooled glycolipids from three mice of each genotype were analyzed by TLC and visualized with orcinol-H_2_SO_4_ as described in *Materials and Methods*. Positions of standard glycolipids are indicated by bars. The bands indicated by an asterisk are not sialic acid-containing glycolipids but presumably a contaminating neutral glycolipid, because of their yellowish color (data not shown).
(TIF)

Figure S2 **Carcinogen-induced ACF formation in** ***Neu3*** **-deficient mice.** Mice were injected with AOM (A) or DMH (B), and the induced ACF were counted as described in *Materials and Methods.*
(TIF)

Figure S3 **A tendency toward reduced susceptibility to colitis-associated colon carcinogenesis in the** ***Neu3*** **-deficient mice.** The *Neu3*-deficient and wild-type mice were subjected to a single AOM injection followed by DSS administration in the drinking water as described in *Materials and Methods*. The tumors were counted by microscopic observation. Vertical bars indicate the mean tumor number. Two independent experiments showed a tendency but not a statistically significant difference (p = 0.119 for exp. 1; p = 0.243 for exp. 2) toward a lower susceptibility to tumorigenesis in *Neu3*-deficient mice than in wild-type mice.
(TIF)
