# Supplementary figures and images for: Reduced Susceptibility to Colitis-Associated Colon Carcinogenesis in Mice Lacking Plasma Membrane-Associated Sialidase
Source: PLoS One. 2012 Jul 17;7(7):e41132. doi: 10.1371/journal.pone.0041132 (PMC3398939; doi:10.1371/journal.pone.0041132)

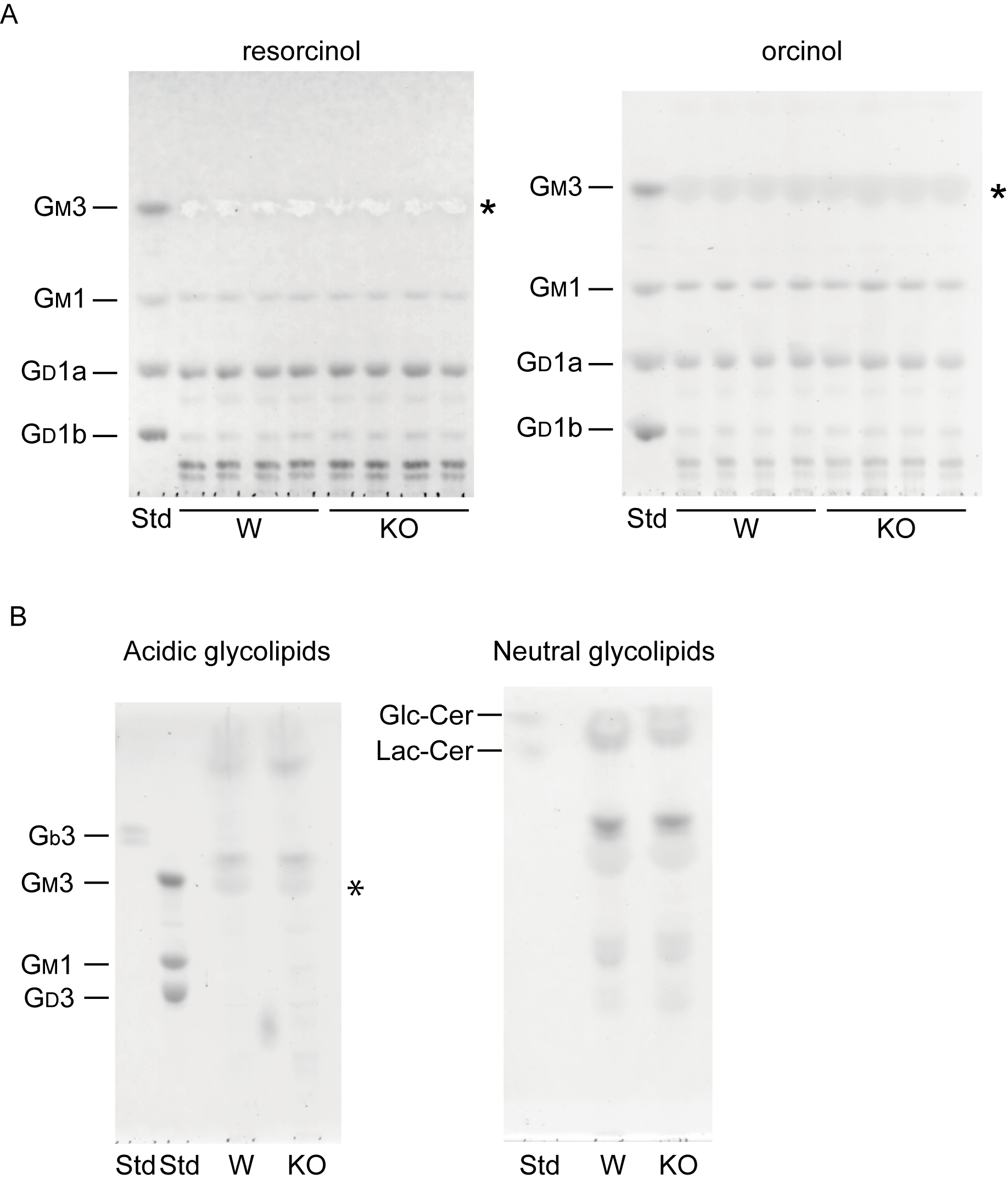

Supplement: Figure S1 — Glycolipid patterns in Neu3 -deficient mice. A. Acidic glycolipids were extracted from the brain of the Neu3-deficient or wild-type mice and analyzed by TLC as described in Materials and Methods. The TLC plates were sprayed with resorcinol-HCl or orcinol-H2SO4 to visualize sialic acid-containing glycolipids or total glycolipids, respectively. Positions of standard gangliosides are indicated by bars. The bands indicated by asterisks were detected by orcinol staining but not by resorcinol staining, suggesting that they were contaminating neutral glycolipids. B. Glycolipids were extracted from the colon mucosa of the Neu3-deficinet and wild-type mice. Pooled glycolipids from three mice of each genotype were analyzed by TLC and visualized with orcinol-H2SO4 as described in Materials and Methods. Positions of standard glycolipids are indicated by bars. The bands indicated by an asterisk are not sialic acid-containing glycolipids but presumably a contaminating neutral glycolipid, because of their yellowish color (data not shown). (TIF) [file pone.0041132.s001.tif]

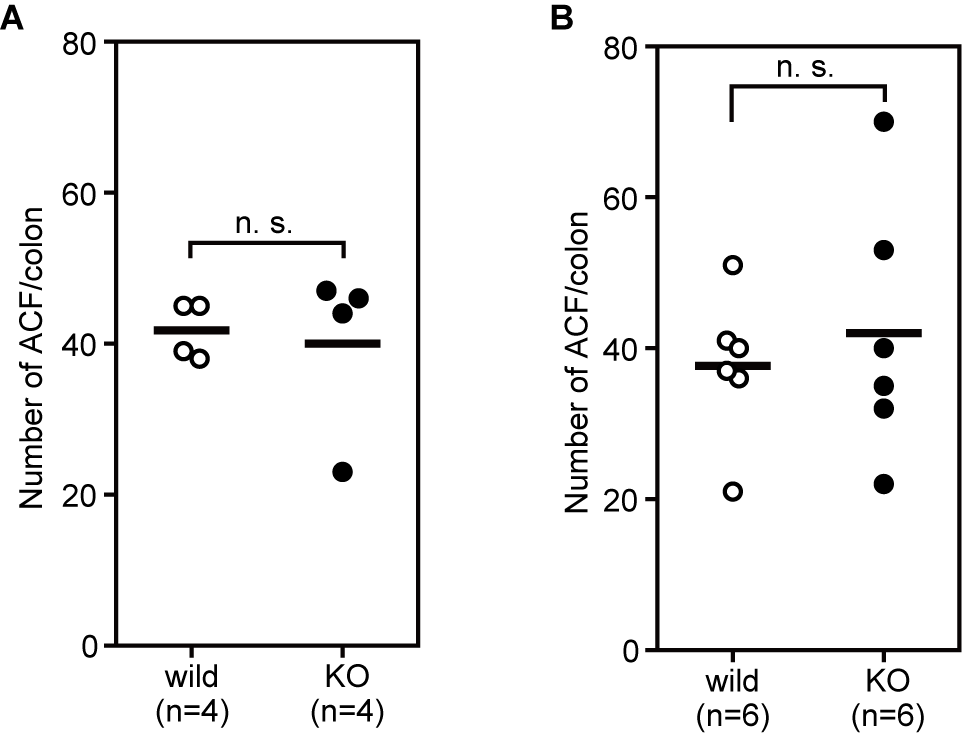

Supplement: Figure S2 — Carcinogen-induced ACF formation in Neu3 -deficient mice. Mice were injected with AOM (A) or DMH (B), and the induced ACF were counted as described in Materials and Methods. (TIF) [file pone.0041132.s002.tif]

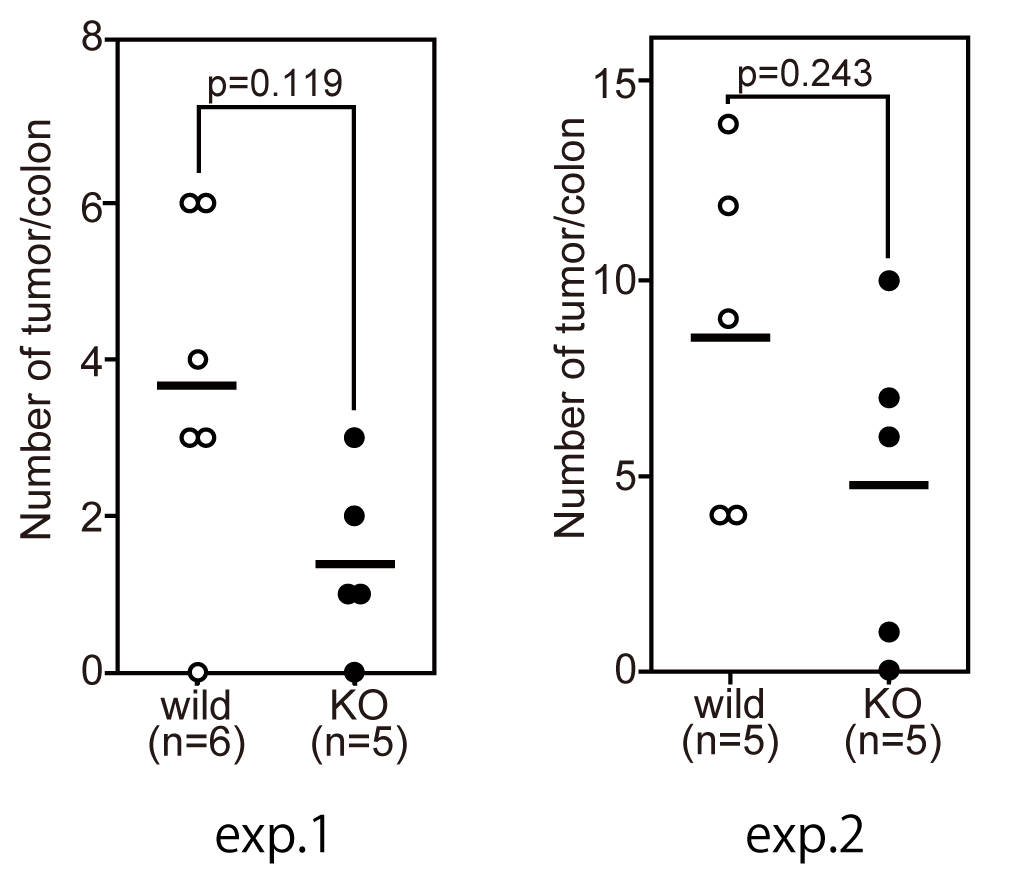

Supplement: Figure S3 — A tendency toward reduced susceptibility to colitis-associated colon carcinogenesis in the Neu3 -deficient mice. The Neu3-deficient and wild-type mice were subjected to a single AOM injection followed by DSS administration in the drinking water as described in Materials and Methods. The tumors were counted by microscopic observation. Vertical bars indicate the mean tumor number. Two independent experiments showed a tendency but not a statistically significant difference (p = 0.119 for exp. 1; p = 0.243 for exp. 2) toward a lower susceptibility to tumorigenesis in Neu3-deficient mice than in wild-type mice. (TIF) [file pone.0041132.s003.tif]
